# The Two-Minute Walk Test in Persons with Multiple Sclerosis: Correlations of Cadence with Free-Living Walking Do Not Support Ecological Validity

**DOI:** 10.3390/ijerph17239044

**Published:** 2020-12-04

**Authors:** Viktoria Karle, Verena Hartung, Keti Ivanovska, Mathias Mäurer, Peter Flachenecker, Klaus Pfeifer, Alexander Tallner

**Affiliations:** 1 Department of Sport Science and Sport, Friedrich-Alexander University Erlangen-Nürnberg, 91058 Erlangen, Germany; verena.vh.hartung@fau.de (V.H.); keti.ivanovska@fau.de (K.I.); klaus.pfeifer@fau.de (K.P.); 2 Department of Education, University of Regensburg, 93040 Regensburg, Germany; 3 Department of Neurology, Klinikum Würzburg Mitte gGmbH Standort Juliusspital, 97070 Würzburg, Germany; m.maeurer@kwm-klinikum.de; 4 Neurological Rehabilitation Center Quellenhof, 75323 Bad Wildbad, Germany; peter.flachenecker@Sana.de

**Keywords:** multiple sclerosis, two-minute walk test, ecological validity, habitual walking, free-living walking, walking performance, accelerometry

## Abstract

The two-minute walk test (2MWT) is a frequently used walking capacity test in persons with multiple sclerosis (pwMS). However, less is known about its relevance with regards to walking capacity during free-living walking performance. Therefore, the ecological validity of the 2MWT was tested by 1. computing free-living minutes with the same intensity (cadence) as during the 2MWT and 2. investigating the relationship between 2MWT cadence and minutes with the same cadence during free-living walking. 20 pwMS aged 44.2 ± 12.2 (Expanded Disability Status Scale (EDSS) score of 3.1 ± 1.4) performed a 2MWT and wore an accelerometer for seven days. The number of pwMS reaching 100%, 90%, 80% or 70% of 2MWT cadence for at least one minute a day and minutes/day with at least 100%, 90%, 80% and 70% of 2MWT cadence during free-living walking was calculated. Six participants reached 100% of the 2MWT cadence for at least one minute/day during free-living walking. A total of 80% 2MWT cadence was the first intensity category that was reached by all participants during free-living walking. No significant correlation was found between cadence in the 2MWT and minutes in which this cadence was reached during free-living walking. Ecological validity with regard to walking intensity could not be confirmed in our study sample.

## 1. Introduction

Multiple Sclerosis (MS) is one of the most common autoimmune neurological diseases and one of the major causes of disability in young adults. Among the most prominent symptoms is disturbed gait. A total of 60% of persons with MS (pwMS) are not fully ambulatory 20 years after disease onset [1]. Furthermore, in a previous study, pwMS rated lower limb function as their most important body function [2]. Consequently, the assessment of ambulation is used to rate disease progression and different objective clinical tools, such as the six-minute walk test (6MWT) [3] and the two-minute walk test (2MWT), have been developed to measure walking capacity under laboratory conditions. The 2MWT is the short version of the 6MWT and was found to provide similar results and validity scores in pwMS, showing no difference in cadence per minute [4,5,6]. Moreover, the 2MWT seems to be more applicable in severely disabled pwMS because it is less time-consuming and exhausting [6]. Still, the 6MWT and 2MWT measure short-term walking capacity as a snapshot of physical functioning. Its association with long-term walking performance in real-life settings, which is an important component of the overall health status of pwMS, is often tacitly assumed but not necessarily existent, since walking behavior in pwMS is subject to a number of physical and behavioral determinants [7]. Therefore, the examination of the ecological validity of laboratory walking measures is highly required in pwMS. Ecological validity is given if a test’s result can be generalized to real-life settings [8]. Even if the main aim of laboratory walking tests is certainly to make statements in a clinical context, examining the meaningfulness of test results for free-living walking performance of patients may offer new opportunities for the usage of the 2MWT e.g., for therapy goal setting, and allow further insights into patients’ walking behavior for practitioners. Free-living walking behavior can be assessed with accelerometers, small activity trackers that are worn directly on the body to measure changes in body acceleration, thus detecting motion. Therefore, accelerometers offer opportunities to compare walking parameters from laboratory tests like the 2MWT to real-life walking. Some studies have already compared the 2MWT performance to overall daily steps [9], length of free-living walking bouts or walking speed [10]. However, since the 2MWT is a measure of walking capacity, which involves walking with higher intensities and speeds, the free-living comparator should also include a dimension for walking intensity. Deducing walking speed from accelerometer signals is a complicated and elaborate procedure, though, and only few research-grade accelerometers are capable of validly assessing walking speed in pwMS [11,12]. Steps per minute or cadence, however, has been shown to be a valid measure for walking intensity in pwMS [13] that can be easily assessed not only by research-grade devices but also with the help of fitness trackers or smartphones. Moreover, as well as distance, which is the most common outcome parameter of the 2MWT, lower cadence was associated with higher disability during the 2MWT [4,14]. Since cadence can be easily assessed both in the 2MWT and through accelerometry, cadence was used as a measure of walking intensity. Consequently, we assume that, at least intra-individually, cadence is a marker for walking intensity.

The goal of this study was to investigate the ecological validity of the 2MWT by comparing cadence (steps per minute) during the 2MWT to activity bouts with comparable cadence gathered from a seven-day accelerometer-based measurement of free-living physical activity. We assumed ecological validity of the 2MWT if (1) the 2MWT intensity (cadence) reflects intensity (minutes with the same cadence) of free-living walking performance, and (2) if higher 2MWT performance is associated with the higher walking capacity found during free-living walking (higher number of minutes with the corresponding 2MWT cadence seen during free-living walking). Taking into account that the 2MWT was actually not designed to reflect free-living walking intensity and is assessed under standardized conditions, we additionally performed an explorative analysis of free-living walking minutes with 90%, 80% and 70% of 2MWT cadence.

## 2. Materials and Methods

Data were gathered as part of the‚ MS bewegt’ study (clinicaltrials.gov NCT04057066). Participants were recruited from one inpatient and one outpatient clinic in Germany through the clinics’ staff or via flyer. During baseline assessment, demographic data including age, gender, height, weight, disease course, duration of disease, Expanded Disability Status Scale (EDSS) score and information about the dominant side were collected and a 2MWT was performed. Patients were asked to wear an ActiGraph wGT3X-BT (Actigraph LLC., Pensacola, FL, USA) accelerometer during the 2MWT and over a seven-day period at home. The ActiGraph was handed out, along with a wear-time diary, which was used to facilitate correct wear-time identification.

### 2.1. Inclusion and Exclusion Criteria

The main inclusion criteria were age ≥ 18 years, MS diagnosis based on McDonald criteria, relapsing–remitting, primary progressive or secondary progressive disease course, EDSS ≤ 6.5, no disease relapse during the last 30 days, and written consent. The main exclusion criteria were severe cardiovascular disease or cortisone therapy during the last 30 days.

### 2.2. Two-Minute Walk Test

The 2MWT measures endurance and functional capacity in ambulation [15] and was found to be reliable and valid in patients with neurological diseases [16] as well as pwMS [5,6,15]. There is no information about psychometric properties when using steps instead of distance as performance outcome in pwMS.

During the test, participants were asked to cover as much distance as possible within a two-minute time frame. The length of pathway was 35m in the outpatient clinic and 50m in the inpatient clinic. Participants were instructed to turn around by walking around cones at the end of the pathway without stopping. PwMS were allowed to use walking aids if necessary and to slow down, stop or take a break if needed. During the test, participants wore a belt with an ActiGraph wGT3X-BT accelerometer on their preferred side on their left or right spina iliaca anterior superior. One tester walked next to the participant, recording distance by using a measuring wheel. Another tester counted steps with a pedometer and stopped time with a stopwatch.

### 2.3. Free-Living PA

Participants were asked to wear the ActiGraph wGT3X-BT under the same instructions as in the 2MWT during waking hours for seven consecutive days at home, and to fill in a printed wear-time diary each day. ActiGraph data were included for further analysis if participants had at least four days with ≥10 h of wear-time, including one weekend day and one weekday according to the guidelines of Gabrys et al. (2015) [17].

### 2.4. ActiGraph wGT3X-BT and Data Processing

Accelerometry has been studied to be an objective method to examine community ambulation in pwMS [18]. Actigraph models from the GT3X series are often used accelerometers in studies on community walking of pwMS and have been found to be appropriate tools in this population [19,20]. In this study, the ActiGraph model wGT3X-BT was used. The ActiGraph wGT3X-BT is a triaxial accelerometer that comes with a proprietary software package (ActiLife, Actigraph LLC., Pensacola, FL, USA) to calculate steps, energy expenditure, physical activity intensity and body position [17,21].

The ActiLife software was configured the same way for the 2MWT and the free-living physical activity (PA) analysis. A sample rate of 100 Hz was used. Raw data (GT3X-files) were transformed into the proprietary AGD format with 60 s bouts using the low-frequency extension (LFE) filter, and then saved in CSV format for further analysis. Troiano algorithm was used for wear-time analysis and, additionally, wear-time diaries were analysed to ensure correct identification of wear and non-wear periods during the seven-day accelerometer measurement.

Mean, standard deviation and range were calculated for descriptive characterization of the sample. Steps of the 2MWT derived from the ActiLife software were divided by two to calculate average steps per minute (cadence). Afterwards, 90%, 80% and 70% of the 2MWT cadence were computed, and minutes with the corresponding cadence were extracted from the seven-day accelerometer measure in Microsoft Excel and described as average number of minutes per valid measurement day. For descriptive statistics, the number of participants reaching 100%, 90%, 80% and 70% of the 2MWT cadence for at least one minute per day was computed along with mean, standard deviation, minimum and maximum number of respective minutes. Scatter plots were made depicting minutes with at least 100%, 90%, 80% or 70% of 2MWT cadence during free-living walking in relation to the step cadence counted by the ActiGraph during the 2MWT. Spearman’s rho was calculated to examine the association between the 2MWT performance and corresponding number of minutes during free-living walking. Analysis was carried out in SPSS Version 25.0 and significance was noted at *p* < 0.05.

### 2.5. Ethical Approval and Written Consent

All participants were properly informed about the study aims and procedures and gave written consent. The study was conducted in accordance with the Declaration of Helsinki and the protocol was approved by the ethical committee of the Baden-Württemberg Federal Chamber of Physicians (F-2018-059, Germany).

## 3. Results

Descriptive statistics of the sample consisting of 20 pwMS are presented in Table 1. All participants provided valid data for 2MWT and free-living physical activity. Mean distance during the 2MWT was 179.6 m, and mean cadence was 114.9 steps per minute. The accuracy of the ActiGraph sensor for step detection during the 2MWT was 98.9 ± 9.0%. In 17 out of 20 participants, the measurement error for step count was below 5%. Compliance during assessment of free-living physical activity was high, with a mean of 6.7 ± 0.9 valid measurement days and of 14H 21M ± 01H 52M daily wear time. Average daily step count was 13,304 ± 4280.

**Figure 1:**
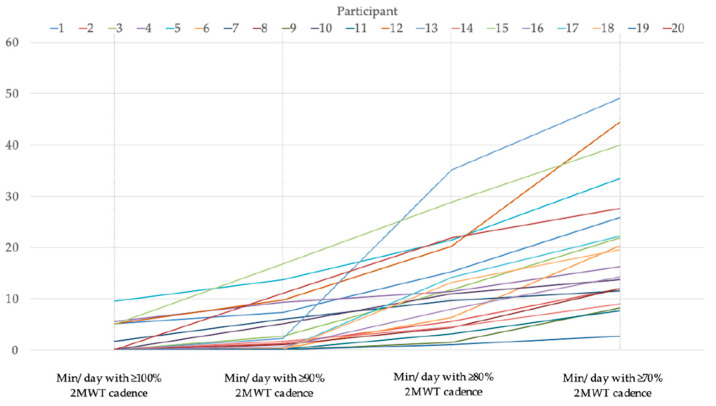
Min/day with at least 100%, 90%, 80% and 70% of 2MWT cadence detected during free-living walking per participant.

Table 2 shows descriptive statistics of the number of participants and free-living walking minutes, with at least 100%, 90%, 80% and 70% of 2MWT cadence. Minutes per day, with 100%, 90%, 80% and 70% of 2MWT cadence walked per participant during free-living walking, are additionally illustrated in Figure 1. Six participants reached 100% or more of the 2MWT cadence for at least one minute per day during walking in daily life, with an average of 5.4 ± 2.5 and a range from 1.7 to 9.6 min. Thirteen participants reached 90% or more of the 2MWT cadence with a mean of 6.8 ± 5.1 min per day, ranging from 1 to 16.9 min. All participants reached 80% or more of their 2MWT cadence at least for one minute a day with a mean of 12.4 ± 9.2 (1–35.1), and 70% or more of their 2MWT cadence with a mean of 20.6 ± 12.8 (2.7–49.1) minutes.

**Figure 2:**
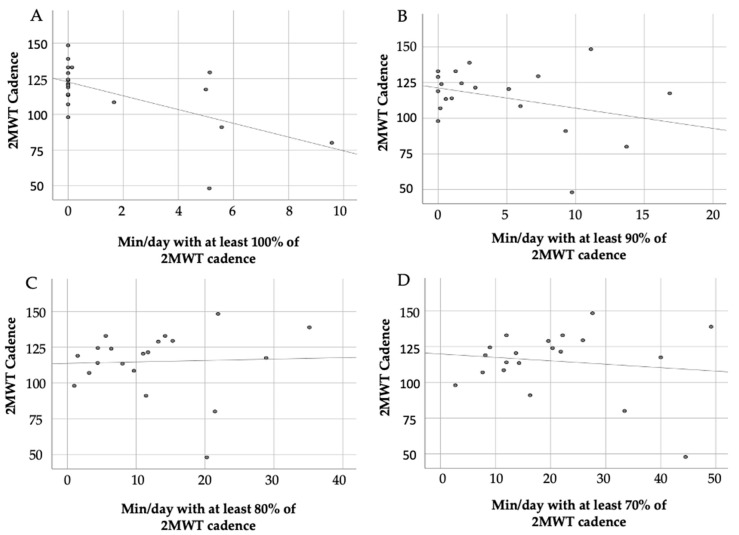
Correlation of 2MWT cadence and minutes in which 2MWT cadence was reached during free-living walking for (**A**) ≥100%, (**B**) ≥90%, (**C**) ≥80% and (**D**) ≥70% of 2MWT cadence.

Scatterplots visualizing the relationship of minutes walked with at least 100%, 90%, 80% and 70% of 2MWT cadence during free-living walking in relation to the 2MWT cadence measured in the clinic are presented in Figure 2A–D. No significant correlation was found when comparing minutes walked with at least 100%, 90%, 80% and 70% of 2MWT cadence during free-living walking and 2MWT cadence from the initial test (Table 3).

## 4. Discussion

The 2MWT is a well-established and meaningful clinical measure of walking function. By examining its ecological validity, we aimed to explore possible new fields of application of the 2MWT results and its possibilities for informing the interpretation of patients’ free-living walking performance by comparing it to free-living walking metrics. Only less than one third of the study population walked with 100% or more of the 2MWT cadence for at least one minute per day during free-living walking. A total of 80% of the 2MWT cadence was the threshold that was reached by all participants, suggesting that walking with intensities of 80% and less of total capacity is more common in free-living walking of pwMS. In addition, no significant correlation was detected between cadence in the 2MWT and corresponding minutes during free-living walking. This was also the case for 90%, 80% and 70% of 2MWT cadence. Based on this, people reaching higher cadences during the 2MWT were not found to also engage in more minutes of intense walking at their individual high-intensity level during free-living physical activity. Thus, participants showing a higher walking capacity during the 2MWT mostly do not make more use of their capacity during free-living walking. Consequently, based on our study sample and in terms of walking capacity, the 2MWT is not able to reflect free-living walking and the ecological validity with regard to walking intensity could not be confirmed. However, it should be noted that the 2MWT measures maximal walking capacity, which is only one influencing factor of free-living walking behavior. Previously, disability level, walking limitations, but also personal factors like physical-activity-related self-efficacy, self-regulation constructs and socio-demographic factors, were observed to be associated with physical activity [7]. In another study, depressed mood and fatigue showed bidirectional links to physical activity [22]. Also, the type of MS (RR, PP, SP) and disease progression could play a role in free-living walking behavior and intensity. Due to our small study sample, sub-group analyses were not feasible, which might have given further insight into the impact of walking capacity on free-living walking performance in different MS groups. In our sample, most of the participants did not reach their walking capacity, found in the 2MWT in free-living walking, at all. No correlation was found between the 2MWT cadence and free-living walking minutes, in which participants reached 100%, 90% 80% and 70% of 2MWT cadence. This may indicate that walking performance during free-living walking was independent from the initially measured 2MWT walking capacity.

In contrast to our results, moderate ecological validity of the 2MWT was found in previous studies. Gijbels et al. (2010) investigated the mean daily stride count and walking distance in the 2MWT and observed significant correlations in the total MS sample and in the subgroup of moderately disabled pwMS, but not in mildly disabled pwMS. The 2MWT was the most predictive variable for habitual walking performance measured through daily stride count in moderately disabled pwMS. Nevertheless, only half of the variances could be explained by the test, indicating that factors not captured by the test are influencing mean daily stride count. However, in their study, walking intensity was not taken into account, neglecting the purpose of the 2MWT to measure walking capacity [9].

Stellmann et al. (2015) noted significant correlations of the 2MWT with different intensity-related parameters (e.g., walking speed in sequences with 50 or 100 consecutive steps) from free-living physical activity and reported the 2MWT to have a moderate ecological validity. Unfortunately, steps per minute were not extracted to determine cadence, which means that their data are not directly comparable to our study data [10].

Engelhard et al. (2018) examined the relationship between the maximum step rate (MSR; highest step rate per minute measured during one-week free-living walking) during free-living walking and the step rate during the 6MWT in pwMS and healthy controls. They found high correlations between the MSR and the 6MWT step rate and determined the MSR to be the best single parameter when measuring walking capacity in free-living walking, therefore being the real-world compliant to the 6MWT. Additionally, habitual walking step rate (HWSR, extracted from activity states that were detected as walking by use of a personalized activity model) together with the MSR were found to be even more efficient, since the MSR tended to overestimate 6MWT step rates and the HWSR had a trend of underestimating them. Engelhard et al. claimed that, in comparison to the MSR and HWSR, conventional habitual physical activity measures like average steps per day are poor measures of walking capacity since they are, in contrast to intensity-based measures, highly influenced by behavioral factors [23].

Despite the correlations found in the above-mentioned studies, no significant correlations were discovered in our study. Our goal was to verify the ecological validity of the 2MWT not only by the calculation of linear associations between global measures, but by determining if the step rate performed during the 2MWT can be also observed during free-living walking. Furthermore, we examined the correlation of the 2MWT step rates and the number of minutes this step rate was reached during free-living walking. By doing so, we wanted to adhere to the original aim of the 2MWT to measure maximal walking capacity. Due to that, we did not compare the 2MWT to general free-living walking measures like steps per day, which also includes gait in low-intensity ranges. By observing 100%, 90% 80% and 70% of 2MWT cadence during free-living walking, we aimed to only target higher intensity walking behavior close to the maximal capacity found in the 2MWT. Thus, our approach to investigate the ecological validity of the 2MWT differed from the mentioned study approaches, explaining the lack of significant correlations in our study.

When rating physical activity intensity, step rate thresholds are usually set on a cohort and not an individual level. For pwMS, Agiovlasitis and Motl (2014) determined step rate thresholds indicating moderate (between 83 and 104, depending upon impairment and height) and vigorous (between 118 and 140) physical activity [13].

With the same moderate physical activity threshold of ≥ 82 steps, Neven et al. (2016) reported that 94% of mildly impaired and 36% of moderately impaired pwMS achieved two uninterrupted minutes at moderate intensity during walking in daily living. At low intensity (step rate < 82), all pwMS reached two and three minutes of uninterrupted walking. In general, people walked 14 ± 14 min per day (1% of daytime) at moderate intensity [24]. This is comparable to our cohort’s average amount of walking minutes at intensities of 70% and 80% of 2MWT cadence. However, no conclusion can be drawn if 70% and 80% of 2MWT cadence in our study are comparable to the applied moderate walking intensity thresholds used by Neven et al. (2016). When those thresholds are applied to the 2MWT in our study, all but two participants exceeded the step rate threshold for moderate physical activity, but only eleven reached the vigorous activity threshold. Claiming that the 2MWT measures maximum capacity, cohort level step rates may not be equally applicable to all individuals with pwMS.

As mentioned above, other influences, such as personal, environmental and behavioral factors or other symptoms [7], may be determinants influencing free-living walking performance. This has implications for clinical interventions. Focusing not only on the improvement of walking capacity during therapeutic interventions, but also addressing behavioral and motivational aspects, could enhance the results of therapy with regards to walking performance in the long run. In addition, the results of our study regarding the grading of the 2MWT cadence in intensity categories might facilitate goal setting and physical activity prescription in interventions. Similar to the prescription of endurance training intensities (calculated as percentage of maximal values assessed during cardiovascular performance diagnostics), the 2MWT cadence might as well serve as a basis for physical activity prescription. Only a few participants reached 100% of their walking capacity, though all walked with 80% of 2MWT cadence for at least one minute per day in daily life. Increasing the number of continuous daily activity minutes with at least 80% of 2MWT cadence might be an achievable and promising goal to elicit health effects in the majority of pwMS. At the same time, prescribing shorter, demanding periods with 100% of 2MWT cadence appears to be promising, as well to improve walking, since walking at faster speeds creates an additional challenge for walking function in pwMS [25].

### 4.1. Limitations

The study demonstrates some limitations which may have influenced the results. First, no standard procedure is available on how to assess wear time periods from the acceleration signals of the ActiGraph. The ActiLife software provides options to choose preset algorithms, and there is evidence about their accuracy depending on a number of parameters [26,27]. Nevertheless, information about the validity of algorithms and configurations for persons with altered gait is scarce. In accordance with the guidelines from Gabrys et al. (2015) [17], we used the Troiano algorithm.

Second, we measured free-living walking in 60-s epochs. Therefore, the steps measured in one minute might have been accomplished within 40 s, followed by a 20 s break. This may dilute the actual walking intensity. To analyse data in shorter epochs of 10 s may be more accurate. However, since many measures are given as number per minute, we wanted to stick to that convention to facilitate calculations and comparability.

Third, even if the ActiGraph was found to be a valid instrument for measuring steps during free-living walking in pwMS [19,20], walking at slow paces, especially in highly disabled pwMS with altered gait patterns [28] or uneven surfaces and short distances during free-living walking, may limit the accuracy of step detection. However, the Actigraph worn on the hip was found to have good accuracy when using the LFE filter for counting steps over short walking bouts in healthy adults [29]. The accuracy of step detection of the ActiGraph in our study was also good during the 2MWT.

Fourth, participants in this study walked, on average, 13,304 steps per day, which is unusually high for the MS population. For pwMS, a recent meta-analysis reports on average 5840 ± 3096 steps per day [30]. After reexamining one dataset, it was evident that the usage of the low-frequency extension filter (LFE) drastically altered the number of steps per day. The LFE aids to detect lower amplitude movements by reducing the internal acceleration threshold that must be crossed before a signal is considered as human movement [31]. In this example dataset, steps per day decreased from 14,146 steps with LFE to 6796 steps without LFE. These data suggest that the LFE filter may have identified false positive steps, and thus presumably has overestimated daily steps. This is in line with findings from other studies that detected the filter to overestimate steps in free-living walking [32,33]. On the contrary, an underestimation of steps per day during free-living ambulation was observed when the LFE was not applied in ActiGraph GT3X models [32,33]. Besides that, the LFE filter is more accurate and suggested in laboratory settings [32], and we wanted to ensure the same data-processing approach in both our laboratory and daily living assessments. Furthermore, the LFE filter only increases sensibility in low-intensity movements. In our sample dataset, the maximum cadence during the entire seven-day measurement changed from 166 with LFE to only 163 without using the LFE. This, taken together with the good accuracy of step detection during the 2MWT in our sample, indicates that our results regarding cadence were not biased by the use of the LFE filter.

Fifth, we determined cadence to be an appropriate measure for walking intensity. However, the relationship between cadence and walking speed in this context could be biased by reduced walking function, especially reduced dynamic stability, in disabled pwMS [25,34]. An increase in walking speed may be accomplished through increased cadence only and not via enlarged step length, since this places high demands on dynamic stability [25]. Therefore, higher cadences in this study may not reflect the same increase in gait speed as in healthy persons. Nevertheless, it was found that moderately impaired MS patients covered less distance, walked slower and had a lower cadence, lower step regularity, lower stride regularity and higher stride time variability in a 6MWT than mildly impaired patients [14]. Motl et al. (2012) also reported less cadence and distance for moderately disabled pwMS compared to mildly disabled pwMS and for highly disabled compared to moderately disabled pwMS during the 6MWT [4]. Thus, not only does step length deteriorate with disability level in pwMS, but also cadence, which both influence distance in timed walking measures. We analysed free-living walking cadence relative to a clinically measured benchmark cadence. It can be assumed that the relationship or change patterns of cadence and step length during continuous walking minutes (as determined by Shema-Shiratzky et al., 2019 [14]) are, intra-individually, rather constant regardless of whether measurements are being performed in clinical or free-living settings. The 2MWT is an accepted measure for walking capacity, but its primary outcome is distance and not cadence. To corroborate our assumptions regarding cadence as intensity measure, further studies investigating the strategies of speed adjustment during continuous walking (step length vs. cadence), depending on disability levels, are needed.

Lastly, we only provide a small study sample of 20 participants, which limits the generalizability of the results.

### 4.2. Implications for Future Research

Further studies with larger samples analysing the ecological validity of the 2MWT with regards to walking intensity are needed. Considering that no relation between the 2MWT and the walking performance in free-living walking with regard to walking capacity could be found, future research should further investigate determinants of free-living walking performance, including type of disease, symptoms and personal and environmental factors. Moreover, possible interactions of those factors with the extent to which walking capacity can be transferred into free-living walking behavior should be analysed. This, in turn, may help to individualise treatment and physical activity promotion interventions. Concerning accelerometer usage, the impact of different wear times and signal sensibility algorithms on the step outcome in populations with altered gait parameters or slow walking speed has to be evaluated.

## 5. Conclusions

The 2MWT was not able to reflect free-living walking performance in terms of walking intensity. Thus, the ecological validity of the 2MWT with regard to walking intensity could not be confirmed in our study sample. Minutes with 100% of 2MWT cadence could only be found in less than a third of pwMS during free-living walking. A total of 80% of 2MWT cadence was reached by all participants, indicating that walking with intensities of 80% and less than total capacity is more common in free-living walking. Other aspects, like disease-related, personal, environmental, behavioral or symptomatic factors, may help to explain free-living walking performance. Future studies could further address the evaluation of determinants of daily living walking performance.

## Figures and Tables

**Table 1:** Mean, standard deviation and range of general characteristics of the sample, accelerometer wear time and descriptive statistics of the 2MWT.

Variable	Sample (*N* = 20)
Age	44.2 ± 12.2 (26–63)
Gender	
female	15 (75%)
male	5 (25%)
EDSS ^1^ (*N* = 18)	3.1 ± 1.4 (1–6)
Duration of disease	9.1 ± 7.7 (1–32)
Disease course	
RRMS ^2^	14 (70%)
SPMS ^3^	4 (20%)
PPMS ^4^	2 (10%)
Free-living PA ^5^	
Measurement days	6.7 ± 0.9
Daily wear time	14H 21M ± 01H 52M
2MWT ^6^	
steps	229.9 ± 45.2 (96–297)
Distance in meter (*N* = 19)	179.6 ± 54.5 (44.1–253.5)
Steps per day	13,304 ± 4280 (6813–20,395)

^1^ Expanded Disability Status Scale; ^2^ Relapsing-Remitting Multiple Sclerosis; ^3^ Secondary-Progressive Multiple Sclerosis; ^4^ Primary-Progressive Multiple Sclerosis; ^5^ Physical activity; ^6^ Two-Minute Walk Test

**Table 2:** Number of pwMS, mean, standard deviation and range of minutes dependent on percentage of 2MWT cadence reached during free-living walking.

Variable	Minutes per Day with at Least % of 2MWT Cadence			
	100%	90%	80%	70%
N	6	13	20	20
Mean	5.4 ± 2.5	6.8 ± 5.1	12.4 ± 9.2	20.6 ± 12.8
Minimum	1.7	1	1	2.7
Maximum	9.6	16.9	35.1	49.1

**Table 3:** Spearman’s rho and *p*-values of the correlation between cadence in the 2MWT and minutes/percentage of 2MWT cadence reached during free-living walking.

Variable *N* = 20	Cadence 2MWT ρ (*p*-Value)
Minutes per day with at least 100% of 2MWT cadence	−0.416 (*p* = 0.068)
Minutes per day with at least 90% of 2MWT cadence	−0.169 (*p* = 0.475)
Minutes per day with at least 80% of 2MWT cadence	0.265 (*p* = 0.276)
Minutes per day with at least 70% of 2MWT cadence	0.224 (*p* = 0.343)
